# Elderly-onset vs adult-onset ulcerative colitis: a different natural history?

**DOI:** 10.1186/s12876-020-01296-x

**Published:** 2020-05-12

**Authors:** Irene Zammarchi, Francesco Lanzarotto, Rosanna Cannatelli, Francesca Munari, Federica Benini, Alessandro Pozzi, Alberto Lanzini, Chiara Ricci

**Affiliations:** 1 Gastroenterology Unit, Spedali Civili Hospital – University of Brescia, Viale Europa, 11, 25123 Brescia, Italy; 2 Gastrointestinal Unit, University of Milan, Milan, Italy; 3 Department of Experimental and Clinical Science, University of Brescia, Brescia, Italy

**Keywords:** Inflammatory bowel disease, Ulcerative colitis, Elderly, Comorbidity, Therapeutic strategies

## Abstract

**Background:**

Incidence of ulcerative colitis (UC) in elderly population is increasing because of ageing and because of its minimal impact on life span. Data on natural history, outcomes and therapeutic strategies are limited.

Our aim is to characterize UC in elderly-onset patients followed at our Inflammatory Bowel Disease outpatient clinic and compare with adult-onset UC.

**Methods:**

From January 2000 to June 2019, 94 patients with UC diagnosed after the age of 65 years (elderly group, E-O) were identified and matched 1–1 according to gender and calendar year of diagnosis with patients diagnosed with UC at age between 40 and 64 years (adult age, A-O).

**Results:**

Comorbidity Index (3.8 vs 1.6, *p < 0.0005*) was higher for elderly UC patients. Symptoms at presentation were similar between the two groups, although abdominal pain was more common in adults, and weight loss was more common in the elderly. At diagnosis, left colitis (61% vs 39%) and proctitis (14% vs 26%) (*p = 0.011*) were more frequent in the elderly. Therapy and clinical behaviour were similar. Surgery was more frequently performed in the elderly (20% vs 9%, *p* = 0.02), while biological therapy was less used (2.1% vs 22%, *p < 0.0005*). Complications were more frequent in the elderly. Extraintestinal manifestations were lower in elderly patients (9.6% vs 19.2%, *p = 0.061*). Time to first relapse was similar between the two groups*.* Mortality (*p < 0.0005*) was higher in elderly patients.

**Conclusions:**

Ulcerative Colitis has similar presentation and behaviour in elderly and adults patients. However, the elderly are more fragile because of comorbidities, increased risk of infections and disease-related complications.

## Background

The number of patients with IBD diagnosed older than 65 years is rising. This is due to the rising incidence of IBD and to an ageing population. Almost 25–35% of IBD patients are ≥60 years old. About 15% of them have been diagnosed during older age; while 20% of them have been diagnosed at a younger age, and now they transitioned into older age [1, 2]. In particular, the incidence of UC varies from 1.1/100.000 to 16.5/100.000/year [3].

There are several differences in clinical presentation, disease course, therapeutic strategies and complications (disease-related and therapy-related) [1, 4–10]. Despite this, clear guidelines on this topic need to be improved, as elderly patients are rarely included in clinical trials.

The aim of our study is to characterize UC diagnosed after the age of 65 years. In particular, our primary aim is to describe clinical presentation of UC in two cohorts of patients: patients who received diagnosis of UC during adult age (particularly between 40 and 64) and patients who received diagnosis of UC when they were 65 or older.

Other goals are to characterize disease course, therapeutic strategies and UC-related and therapy-related complication.

## Methods

For this study we extracted information from a prospectively maintained data base. This included all patients followed at our IBD outpatient clinic (Gastroenterology Unit, Spedali Civili Hospital, Brescia, Italy) diagnosed with IBD from 1th January 2000 to 30 June 2019. Ninety-four patients with UC diagnosed after the age of 65 years (elderly group, E-O) were identified and matched 1–1 according to gender and calendar year of diagnosis with patients diagnosed with UC at age between 40 and 64 years (adult age, A-O). This study design was chosen, at the expense of sample size, to control for factors potentially affecting comparisons between the 2 study group. Firstly because gender related differences in clinical presentation, disease location and natural history are known to occur in UC [11]. Secondly because during a very long period of enrollment (20 years) changes in diagnostic methods, therapeutic options, disease awareness, referral rates and possibly also environmental factors are likely to occur providing a rationale for matching patients for calendar year of diagnosis.

The only exclusion criteria was the loss of the patient at follow up (6 pts), because lack of information related to surgery, new therapies, complication, adverse events, infections might lead to unrepresentative conclusions.

The following data were collected: personal data (sex, birth year, age at diagnosis), risk factors (appendectomy, family history, smoking), disease extent according to Montreal Classification, symptoms at the time of diagnosis, time between symptoms onset and diagnosis, misdiagnosis, clinical disease activity, anaemia, extraintestinal manifestations, non-UC medical treatment at the time of diagnosis, comorbidity index and severity index of CIRS (Cumulative Illness Rating Scale), therapy for induction and maintenance of remission, time to first relapse, surgery, UC-related and therapy-related complications.

### Statistical analysis

Qualitative variables were expressed as numbers and percentage, while quantitative variables were expressed as mean value, median and standard deviation or interquartile range. Qualitative variables were analyzed using Fisher Exact Test or Chi-squared statistics between the two groups. Quantitative variables were analyzed using T-test for non-matched couples or Mann-Whitney test as appropriate. A value of *p < 0.05* was considered statistically significant.

Data was transferred to a Microsoft Excel database and exported to Stata version 14 (StataCorp LLC, Texas, USA).

Ethical approval for this study was granted by the Brescia Province Ethics Committee, Spedali Civili of Brescia (10/21/2016) and informed consent was obtained from all patients.

## Results

### Demographic information and risk factors

94 patients who have been diagnosed with UC after the age of 65 years were compared with 94 patients who have been diagnosed with UC between 40 and 64 years. The demographic characteristics of both group are shown in Table 1. Median age at diagnosis was 71.5 ± 5 years and 50.1 ± 6.7 years in E-O and A-O, respectively (*p < 0.0005*). Male patients were more frequent in both groups (54%). Family history for UC was similar in the two groups (9,4% vs 8,4%, *p = 0.824*). Difference in smoking habits, althoughs not statistically significant (*p = 0.150*) was reported. Time between onset symptoms and UC diagnosis was similar in the two groups, 5.8 ± 4.6 months in the elderly and 6.2 ± 4.7 months in adults (*p = 0.5872*) respectively. 34% of the elderly and 19% of the adults received a different diagnosis before UC diagnosis. The most frequent one was diverticular disease in the first group (15%), while infectious colitis was the most common in the second group (9%).

**Table 1:** Demographic characteristics of UC by age

Characteristics	Elderly (≥ 65 yrs) ***N*** = 94	Adults (40–64 yrs) ***N*** = 94	***p value***
Median age at diagnosis (yrs, SD)	71.5 (5.0)	50.1 (6.7)	*< 0.0005*
Female N (%)	43 (45.7)	43 (45.7)	*0.883*
Family history for IBD N (%)	8 (9.4)	7 (8.4)	*0.824*
Smokers N (%)	7 (8.5)	13 (15.3)	
Ex smokers N (%)	34 (41.5)	41 (48.2)	*0.150*
Non smokers N (%)	41 (50.0)	32 (36.5)	
Follow up lenght, yrs. mean (SD)	8.1 (15.6)	5.7 (13.2)	*0.1155*
Time from symptoms onset to diagnosis, yrs., mean SD	5.8 (4.6)	6.2 (4.7)	*0.5872*
Comorbidity Index (CIRS)	3.78 (2.02)	1.57 (0.87)	*0.0005*

Median follow among elderly patients up was 8.1 years and 5.7 years in the control group. Mortality was 20.5% (18 pts) in the elderly and 3.9% (3 pts) in adults. *p < 0.0005*. Main causes of death were cardiovascular disease (39%), sepsis (22%), extraintestinal neoplasia (17%) and respiratory failure (17%) in the elderly; cardiovascular disease (34%), sepsis (33%) and extraintestinal neoplasia (33%) in adults.

### Clinical presentation at diagnosis

**Fig. 1:**
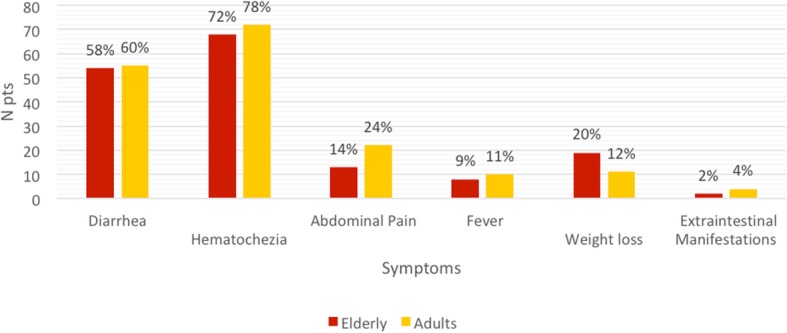
Different symptoms at UC onset in both groups

Some differences were found in the two groups, although none of them was statistically significant. (Fig. 1). Abdominal pain was less common in the elderly (14% vs 24%), while anaemia (52% vs 39%) and weight loss (20% vs 12%) were more common. No differences were found for diarrhoea (58% vs 60%), hematochezia (72% vs 78%), fever (9% vs 11%) and extraintestinal manifestations (2% vs 4%).

### Multimorbidity and polypharmacy

Multimorbidity (presence of 2 chronic disease or more) was evaluated with CIRS (Cumulative Illness Rating Scale), and it was more common in the elderly.

At IBD diagnosis, median value of comorbidity index was 3.8 for the elderly and 1.6 in the adults (*p < 0.0005*), while median value of severity index was 1.6 and 1.3, respectively (*p < 0.0005*).

History of myocardial infarction (21% vs 3%, *p < 0.0005*), stroke (8%, *p 0.007*) and malignancy (10% vs 4%, *p 0.151*) was more common in the elderly.

Most of elderly patients (66%) had 3 or more chronic diseases, while most of adults (62%) had only one chronic disease (*p < 0.0005*). Multimorbility was found in 89% of the elderly and 36% of the adults (*p < 0.0005*).

Several differences in number of drugs taken at the time of diagnosis between the two groups were reported. Median value was 3 in the elderly and 1 in the control group (*p < 0.0005*). 42% of elderly patients used from 2 to 4 drugs, while 82% of adults used just one drug.

### Disease extent and phenotype at the time of diagnosis and at follow up

**Fig. 2:**
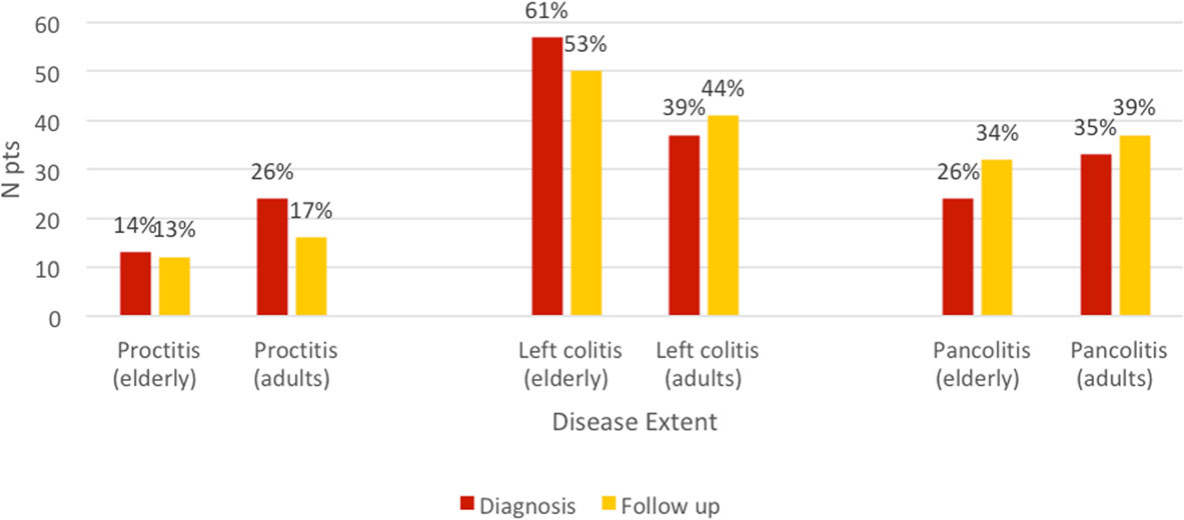
UC extent at diagnosis and at final follow up in both groups

Disease extent was compared in the two groups and within each group, both at the beginning and at maximum follow up. (Fig. 2).

At diagnosis, left colitis was more common in the elderly (61% vs 39%), while proctitis (14% vs 26%) and extensive colitis (26% vs 35%), *p 0.011*) were less common.

During follow up, a variation in the disease extent was found in both groups (14% of the elderly and 12% of the adults) (*p 0.402*).

### Disease course, therapeutic strategies and complications

The use of mesalamine as a therapy for induction of remission was similar in the two groups (58.5% vs 54.8%), as well as steroid therapy (38.3% vs 33.3%) and surgery (5.3% vs 3.2%) (*p 0.479*). (Table 2).

**Table 2:** UC therapeutic strategies, disease course and complications

	Elderly (≥ 65 yrs) ***N*** = 94	Adults (40–64 yrs) ***N*** = 94	***p value***
**Therapy at the diagnosis**			
Mesalamine N (yrs, SD)	55 (58.5)	51 (54.8)	*0.612*
Steroids N (%)	36 (38.3)	31 (33.3)	*0.479*
Surgery at the diagnosis (%)	5 (5.3)	3 (3.2)	*0.479*
**Follow-up**			
Surgery N (%)	19 (20.4)	8 (8.5)	*0.020*
Months to surgery, mean (SD)	18.4 (34.1)	24,87 (23.9)	*0.2903*
Mesalamine N (%)	90 (95.7)	86 (96.7)	*0.351*
Immunosuppressant N (%)	10 (10.8)	11 (12.1)	*0.837*
Biological therapy N (%)	2 (2.1)	20 (22)	*< 0.0005*
Patients with one relapse or more, N (%)	54 (57.5)	52 (57.1)	*0.932*
Time to first relapse in months, mean (SD)	19.04 (28.6)	16,49 (22.9)	*0.5615*
Extraintestinal manifestations N(%)	9 (9.6)	18 (19.2)	*0.061*
**Complications**			
Intestinal complications N (%)	17 (18.1)	5 (5.5)	*0.0007*
Systemic infections, N (%)	28 (29.8)	24 (25.5)	*0.514*

Globally, surgery was performed more frequently in the elderly (20.4% vs 8.5%) (*p 0.020*), with a mean time between diagnosis and surgery of 18.4 months in the elderly and 24.87 months in younger patients (*p 0.2903*).

The type of surgery most frequently performed was partial colic resection (41,2%), followed by perianal disease (29,4%), colectomy (17,6%) and resection for cancer (11,8%) in the elderly; colectomy (87,5%) and perianal disease (12,5%) in adults.

As maintenance therapy, there was no difference between the two groups in the use of mesalamine (95.7% vs 96.7%) and immunosuppressant (10.8% vs 12.1%); while biologic agents (antiTNFα and anti-α_4_β_7_ integrin) were much less used in the elderly (2.1% vs 22%) (*p < 0.0005*).

During follow up, 57% of patients had at least one disease exacerbation, with a similar median time to first relapse (19.04 months in the elderly and 16.49 in the control group). *p 0.5615*.

Extraintestinal manifestations were less common in the elderly (9.6% vs 19.2%), (*p 0.061*). Intestinal complications were more frequent in the elderly (18.1% vs 5.5%, *p 0.0007*). In particular, stenosis was the most frequent complication in both groups. The other ones were toxic megacolon, intestinal perforation and haemoperitoneum.

Moreover, major infections were more common in the elderly (29.8% vs 25.5%) (*p 0.514*). The most frequent infection was pneumonia in both groups (15% vs 6%). The other ones were sepsis (8% vs 4%), *C. difficile* infection for the elderly (5% vs 1%) and CMV infection for the adults (4% vs 5%). Herpes Zoster infection and systemic candidiasis were also reported.

### Iatrogenic complications, adverse drug reactions and new pathologies

Despite the similar use of steroid therapy in the two groups, iatrogenic complications were more common in the elderly (18% vs 11%). In particular, the most frequent ones were steroid diabetes (8% vs 4%) and osteoporosis (11% vs 5%).

**Fig. 3:**
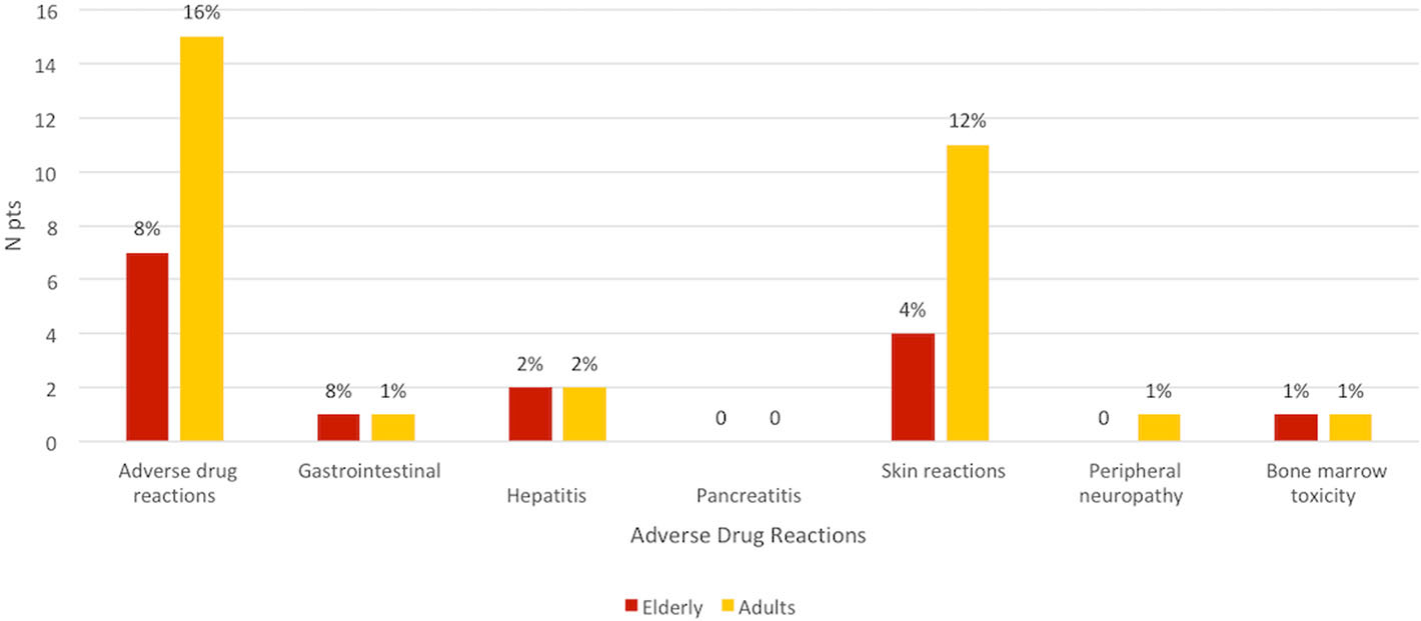
Adverse drug reactions during follow up (*p* < 0.0005)

Unexpectedly, adverse drug reaction were less common in the elderly (8% vs 16%, *p < 0,0005*). (Fig. 3). However, if we exclude patients on biological therapy, they were similar in the two groups (8% in the elderly and 9% in the control group). Globally, 10 adverse drug reactions developed in the elderly and 16 in the adult group, and the most frequent ones were skin reactions.

During follow up, some conditions typical of old age were considered. A higher number of elderly patients developed cognitive impairment (15% vs 1%) (*p < 0.0005*). Malignancy (15% vs 10%), deep vein thrombosis/pulmonary embolism (15% vs 4%), myocardial infarction (10% vs 1%), stroke (3% vs 1%) and depression (14% vs 13%) were also more frequent (ns).

## Discussion

In this study we analyzed and we compared clinical presentation, disease course, complications and therapeutic strategies of UC in a group of patients who were diagnosed with UC during old age (≥ 65 years) and in a group of patients who were diagnosed with UC between 40 and 64 years. The study is prospective observational, and the two groups are homogeneous for type of disease, sex and age at diagnosis.

Among the patients followed at our IBD outpatient clinic (Gastroenterology Unit, Spedali Civili Hospital), from 1° January 2000 to 30 June 2019 all new diagnoses of UC were considered. The total study number of patients was 188, 94 elderly and 94 adults. A median follow up of 7 years was reached.

Family history, smoking habits and NSAIDs use are confirmed as risk factors for UC. In fact, a family history for UC (< 10%) was found in both groups among 1st grade relatives, as we can see in the EPIMAD register (IBD register in North-western Italy). In line with previous studies [12], non smokers or ex smokers are prevalent in UC (*p 0.002*).

Time between symptoms onset and UC diagnosis is similar between the two groups, with a median value of 6 months. The most common misdiagnosis was diverticular disease, probably because the prevalence of diverticula was significantly higher in the elderly (*p < 0.0005*).

Compared to previous studies [13–15], diagnostic delay was higher in the elderly.

Data about clinical presentation and disease extent are similar to the ones we find in previous studies [15–22], with differences between elderly and adults not statistically significant.

UC clinical presentation was similar in the two groups, except for abdominal pain, which was more common in the elderly. At UC diagnosis, left colitis was more common in the elderly, while proctitis was less common (p 0.001). During follow up, 13% of patients had an extension of the disease [15, 17, 20–22].

Data about extraintestinal manifestations are similar to the ones we find in earlier studies [13, 14]. Although at the time of diagnosis the number of patients with extraintestinal manifestations was similar in both groups, during follow up they were less common in the elderly (*p 0.06*). There may be several reasons for these differences.

Most of IBD extraintestinal manifestations involve joints. Joint pain in the elderly may be classified as a pain due to arthrosis; patients may not refer this kind of pain, because they consider it as a part of geriatric disease. Moreover, most of extraintestinal manifestation, in particular the arthritic ones, develop during disease exacerbation. Disease relapses are less frequent in the elderly and this could explain why extraintestinal manifestations develop less frequently.

According to previous studies [18, 23, 24], many differences were found also in therapeutic strategies. Therapy for induction of remission was similar in the two groups, with a similar use of mesalamine and steroids [25].

Many differences were found for surgery. The use of surgery for the induction of remission was similar in the two groups, while its use during follow up was much more frequent in the elderly (*p 0.02*). Thus, surgery is performed more frequently in the elderly. This may be due to limitations for some medical therapies during geriatric age. In fact, the number of patients who received immunomodulating therapy was significantly lower [19, 26–29].

Limitations in medical therapy are due to multimorbidity and polypharmacy typical of geriatric age [30]. In fact, at the time of diagnosis elderly patients have a comorbidity and severity index significantly higher compared to adult population (*p < 0,0005*) and, subsequently, a higher number of drugs taken (*p < 0.005*). At the time of diagnosis, many elderly patients had a history of cancer, myocardial infarction and heart failure. These diseases contraindicate a therapy with immunosuppressant and antiTNFα.

As widely reported in literature [24, 31–34], disease-related complications and therapy-related complications are more frequent in the elderly. Frequency of intestinal complications, infectious complication and side effects of steroid therapy is higher, while frequency of adverse drug reactions is similar in the two groups, if we exclude the ones related to biological therapy.

The most frequent intestinal complications were stenosis. However, they all take place in patients who also have a concomitant diverticular disease. The most frequent infectious complications were pneumonia, sepsis, complicated UTI, while the most relevant side effects of steroid therapy were diabetes, osteoporosis and hypertension [35–38].

Multimorbidity and polypharmacy may be relevant for the development of these complications. Intestinal complications may be due to a poor control of the disease deriving from polypharmacy, which determines, in the first place, a lower adherence to therapy. This may be worsened by cognitive impairment [39, 40], which was found in 18% of patients at the end of follow up. Secondly, even when the adherence to therapy is adequate, elderly patients have alterations in all levels of pharmacokinetics, with altered absorption (achlorhydria), distribution (hypoalbuminemia and dehydration), metabolism (enzyme induction) and excretion (kidney failure). These elements may create interactions which are often unknown or not characterisable, and may determine a major or minor drug activity with intraindividual and interindividual variability.

Alternatively, if we consider the major frequency of side effects related to steroid therapy, the reasons are similar to the ones previously described for intestinal complications. Moreover, this kind of therapy may worsen other conditions which are frequent during geriatric age, such as osteoporosis, diabetes, hypertension, insomnia and major infectious risk, which can be also related to the senescence immune system of the elderly.

**Fig. 4:**
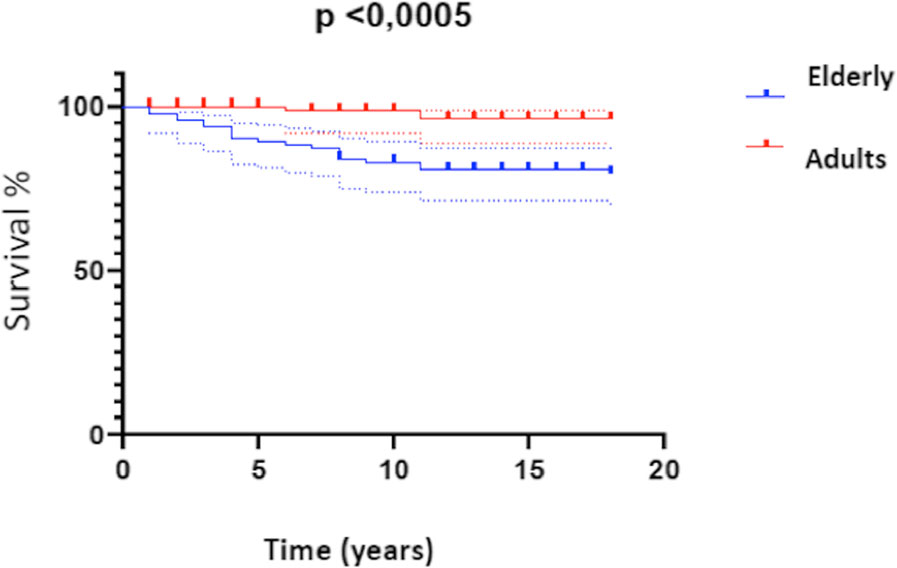
Kaplan-Meier graphic for mortality in UC during the follow-up

Finally, considering incident pathologies during follow up, we can find more frequently cognitive impairment, DVT/pulmonary embolism, myocardial infarction, stoke and cancer, both intestinal and non intestinal. Moreover, mortality is significantly higher during geriatric age (20% vs 2%, *p < 0.0005*). (Fig. 4)

## Conclusions

In this prospective study comparing UC diagnosed during geriatric age and during adult age (40–64 years) risk factors are similar between the two groups, although they present small variations. Clinical presentation and disease extent present variation that need to be taken in account. However, the greatest differences are represented by multimorbidity, therapeutic strategies (medical and surgical) and complications. Multimorbidity limits medical therapy. This determines a major number of complications that can’t be treated with medical therapy, and they require more frequently surgical therapy. Moreover, even when a medical therapy is possible, this may lead to significant side effects and infectious complications.

Thus, an assessment of the benefit-risk ratio is required. The elderly patient, as it is a more fragile patient, needs to be re-evaluated considering new pathologies during follow up, making several therapeutic changes.

The high number of patients involved in this study allows a more precise characterization of UC during old age in a setting where, frequently, the patients who are subjects of study are reduced, basically because of issues surrounding the management of the elderly in clinical trials. This represented, for a long time, a problem in the patient management with IBD, because without proper studies we cannot derive improved guidelines.

## Data Availability

The datasets used and/or analysed during the current study are available from the corresponding author on reasonable request.

## Abbreviations

A-O: Adult onset
E-O: Elderly onset
IBD: Inflammatory bowel disease
UC: Ulcerative colitis
CIRS: Cumulative illness rating scal
